# Microbial communities in different regions of the gastrointestinal tract in East Asian finless porpoises (*Neophocaena asiaeorientalis sunameri*)

**DOI:** 10.1038/s41598-018-32512-0

**Published:** 2018-09-20

**Authors:** Xiao-Ling Wan, Richard William McLaughlin, Jin-Song Zheng, Yu-Jiang Hao, Fei Fan, Ren-Mao Tian, Ding Wang

**Affiliations:** 1 The Key Laboratory of Aquatic Biodiversity and Conservation of the Chinese Academy of Sciences, Institute of Hydrobiology, Chinese Academy of Sciences, Wuhan, 430072 China; 2 University of Chinese Academy of Sciences, Beijing, 100039 China; 3 General Studies, Gateway Technical College, Kenosha, WI 53144 USA; 4 Institute for Environmental Genomics, University of Oklahoma, Norman, OK 73019 USA

## Abstract

Mammalian gastrointestinal (GI) tract microbial communities are critical for host health. However, the microbiota along the GI tract in cetaceans has not been well characterized compared to other animals. In this study, the bacteria and fungi present in the stomach, foregut, hindgut and feces, of East Asian finless porpoises (*Neophocaena asiaeorientalis sunameri*, EAFPs) were characterized using high-throughput sequencing analysis. The bacterial and fungal diversity and richness in the stomach, hindgut and fecal samples tended to be higher than those in the foregut. Bacterial taxonomic compositions found in the hindgut and feces were different from those seen in the stomach and foregut. A greater proportion of strict anaerobic bacteria including Clostridia, Fusobacteria, and Ruminococcaceae were found in the hindgut and fecal samples. The fungal communities present in stomach samples differed from those detected in other regions to some extent. Zygomycota and Neocallimastigomycota were more predominant in the stomach. Some potential pathogens, such as *Helicobacter* spp. and *Vibrio* spp., were commonly present along the GI tract. Our study confirms that the fecal microbiota can represent the whole GI tract to some extent because of their relatively higher microbial diversity and presence of potential pathogens. Our study provides the first comprehensive characterization of the EAFPs GI microbiota, expanding on the current knowledge about the bacterial diversity in the GI tract of cetaceans. In addition, this is the first study characterizing the fungal diversity of any species of porpoise.

## Introduction

The adult human’s gastrointestinal (GI) tract harbors various microorganisms which are more numerous than the total number of mammalian cells^1^. The GI microbiota plays a crucial role in many important physiological processes of the host, such as nutrition absorption, development of disease and maintaining good health^2,3^. In addition, these microbes have also been proved essential in the host-microbes evolutionary process^4^. And thus, comprehensive characterization of GI microbiota commonly found in hosts is central to better understanding and predicting relationships between microorganisms and hosts. Recently, GI microbial studies from a diverse range of mammals have been substantially benefited from culture-independent high-throughput sequencing techniques^5^. Studies focusing on the gut microbiota of both humans^6–8^ and other terrestrial animals^9–11^ have been extensive. However, there has been relatively little research on vulnerable cetaceans, an essential part of marine ecosystems as sentinel or indicator species for ecosystem health and integrity^12^. This is probably due to the difficulty in obtaining samples^13–15^.

In previous studies, fecal samples were used to determine the microbiota present in the GI tract of cetaceans^13–17^. This was mainly due to the ease of sample collection compared to the invasive procedures required to collect samples from other GI regions. However, the mammalian digestive tract consists of different compartments with distinct physicochemical conditions, which help select for different microbiota that are adaptive to the specific anatomical site^18,19^. In this study we are interested in determining what is the microbial composition along different regions of the cetacean GI tract and if the fecal microbiota is a good index of the true GI tract microbial ecosystem in cetaceans. We hypothesized that the different regions of the cetacean GI tract may harbor a distinct microbiota.

In the present paper, we characterized the bacterial and fungal communities present in the stomach (forestomach), foregut, hindgut, and rectum (feces) of East Asian finless porpoises (*Neophocaena asiaeorientalis sunameri*, EAFPs). EAFPs are one of two subspecies of narrow-ridged finless porpoises (*N*. *asiaeorientalis*)^20^ and widely distributed in coastal waters from the Taiwan Strait to the Bohai/Yellow Sea in China, and in waters off the coast of Korea and Japan^20^. EAFPs have suffered a population decline and they are now classified worldwide as Vulnerable (VU) according to the IUCN Red List of Threatened Species^21^. In spite of their status, knowledge of their GI microbiota is scarce. The fungi present in the GI tract in cetaceans is quite limited and has only been reported recently in two blue whales (*Balaenoptera musculus*)^14^. And thus, our study also involved the fungal analysis in the EAFP GI tract. To our knowledge, the present work is the first study on the microbiota along the GI tract of a cetacean species. This work will aid in providing baseline information about the microbiota present in the GI tract of other cetaceans.

## Results

### High-throughput sequencing for bacterial and fungal community in the EAFP GI tract

**Figure 1:**
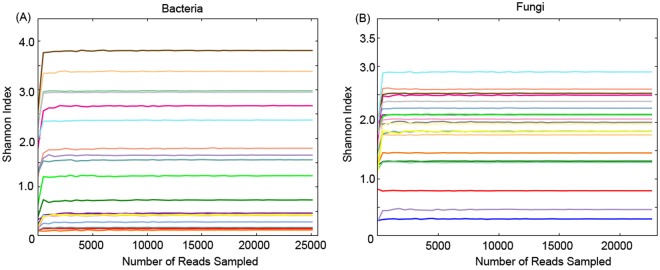
Rarefaction curves of Shannon diversity analysis of all the bacterial (**A**) and fungal (**B**) samples of 5 East Asian finless porpoises sampled in the study.

A total of 700,999 sequences (ranging from 25,154 to 44,511 sequences per sample) were obtained for bacteria in all the 20 samples from the 5 EAFPs, with an average of 35,050 sequences per sample. As for fungi, a total of 664,359 sequences (ranging from 22,655 to 40,505 sequences per sample) were obtained, with an average of 33,224 sequences per sample. To normalize the data prior to data analysis, resampling depth at 25,150 and 22,650 sequences was performed for bacterial and fungal analysis, respectively. In total, 179 bacterial operational taxonomic units (OTUs) were identified at 97% sequence similarity, while less OTUs (91 OTUs) were identified for the fungal community. Rarefaction curves of the Shannon diversity tended to reach a plateau at the current resampling sequencing depth for all the bacterial and fungal samples (Fig. 1). Moreover, the Good’s coverage (bacterial and fungal) of each sample was above 99.9%. This suggested that the current resampling sequencing depth was deep enough to represent most of the microbial diversity. Microbial richness (number of OTUs and Chao 1) and diversity (Shannon indices) are presented in Table 1.

**Table 1:** Summary of number of OTUs, Chao 1 richness and Shannon diversity indices (average ± standard error) of the bacteria and fungi present along the GI tract and each individual East Asian finless porpoises.

	Bacteria			Fungi		
	No. of OTUs	Chao1	Shannon	No. of OTUs	Chao1	Shannon
**Region**						
Stomach	129	62.07 ± 9.14	2.13 ± 0.49	35	8.60 ± 1.63	1.71 ± 0.38
Foregut	106	55.72 ± 13.60	0.38 ± 0.11	15	3.60 ± 0.40^*^	1.20 ± 0.31
Hindgut	150	62.10 ± 14.39	1.36 ± 0.59	36	9.00 ± 1.90	2.15 ± 0.13
Feces	121	55.97 ± 7.80	1.71 ± 0.64	43	9.80 ± 1.74	2.23 ± 0.21
**Subject**						
Subject 1	50	26.80 ± 5.79	0.92 ± 0.69	28	8.50 ± 2.90	1.48 ± 0.28
Subject 2	96	65.12 ± 5.42	1.42 ± 0.40	33	9.50 ± 2.75	1.44 ± 0.64
Subject 3	112	66.39 ± 15.26	0.68 ± 0.38	19	6.50 ± 1.44	2.02 ± 0.23
Subject 4	92	66.31 ± 4.13	1.38 ± 0.59	21	5.75 ± 0.95	1.94 ± 0.24
Subject 5	123	70.22 ± 12.70	2.58 ± 0.74	27	8.50 ± 1.85	2.23 ± 0.17

*Significance *P* < 0.05 (pairwise wilcoxon rank sum test).

### Changes in bacterial and fungal community diversity and structure along the EAFP GI tract

Generally, the richness was relatively even across all the sites as determined by the Chao 1 index for the bacterial samples (Table 1), while for fungal samples the richness was significant lower in the foregut than in the stomach, hindgut and feces (Table 1). However, in both bacterial and fungal samples, the diversity was lower in the foregut than in the stomach, hindgut and feces, as determined by the Shannon index (Table 1). In addition, both the bacterial and the fungal diversity (indicated by Shannon index), and the fungal richness (indicated by number of OTUs and Chao 1 index) increased along the intestinal tract (from the foregut to feces) (Table 1).

**Figure 2:**
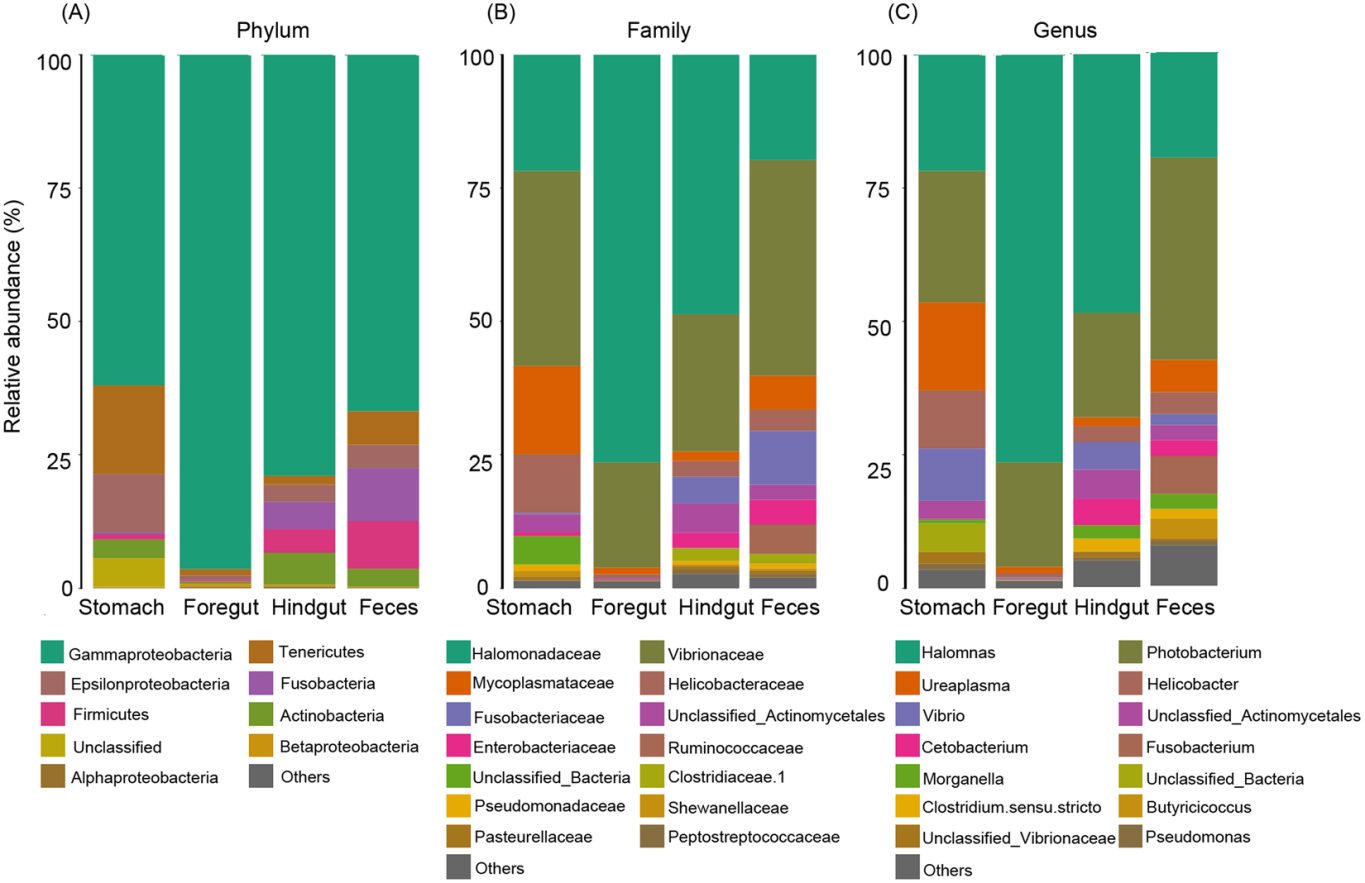
Bacterial compositions along the GI tract of East Asian finless porpoises at the Phylum (**A**), Family (**B**) and Genus (**C**) levels.

Thirteen bacterial phyla were identified along the EAFP GI tract. At deeper taxonomic levels, 38 orders, 66 families and 114 genera were identified. Abundances of major phyla differed along the GI tract (Fig. 2A). Gammaproteobacteria (Mean ± SE, 76.02 ± 5.94%) was dominant along the GI tract, followed by Tenericutes (6.44 ± 2.82%), Epsilonproteobacteria (4.82 ± 1.75%), Fusobacteria (3.87 ± 2.12%), Firmicutes (3.64 ± 2.22%), Actinobacteria (3.33 ± 1.30%), Betaproteobacteria (0.26 ± 0.10%), Alphaproteobacteria (0.19 ± 0.04%) and Bacteroidetes (0.04 ± 0.02%). The remaining identifiable phyla and the unclassified bacteria were detected at a relatively low abundance <2%. Among these phyla, only Gammaproteobacteria and Actinobacteria were identified in all samples. Tenericutes was more abundant in the stomach (16.55 ± 7.33%) than in the feces (6.32 ± 5.48%), hindgut (1.70 ± 1.27%) and foregut (1.20 ± 1.03%). The relative abundance of Fusobacteria and Firmicutes present in the feces (9.98 ± 6.66% and 8.91 ± 8.04%, respectively) and the hindgut (5.11 ± 4.97% and 4.43 ± 4.06%, respectively) were higher than those in the stomach and foregut. A small percentage of Actinobacteria was detected in the stomach (3.53 ± 1.74%), hindgut (5.97 ± 4.27%), feces (3.37 ± 2.38%) and foregut (0.44 ± 0.14%).

At the family level, the top fifteen most abundant groups are showed in Fig. 2B. Halomonadaceae (41.63 ± 8.96%) and Vibrionaceae (30.66 ± 9.28%), both belonging to the phylum Gammaproteobacteria, dominated the GI tract. Mycoplasmataceae (16.55 ± 8.96%) and Helicobacteraceae (10.91 ± 4.97%) were also prevalent in the stomach but were less in the foregut, hindgut and feces. Fusobacteriaceae was present in the feces (9.98 ± 6.66%) and hindgut (5.01 ± 5.00%) but was detected at <2% in the other two sites. It was noted that the Ruminococcaceae (belonging to the Firmicutes) present in the feces (5.52 ± 4.79%) was detected at a significantly higher level than that in the stomach (*P* = 0.039) and foregut (*P* = 0.039).

At the genus level (Fig. 2C), the unclassified members from the family Ruminococcaceae was detected at significantly higher level in the feces (1.76 ± 1.14%), compared with the stomach (*P* = 0.019), foregut (*P* = 0.019) and hindgut (*P* = 0.043). *Halomonas* was the most prevalent genus along the GI tract (41.63 ± 8.96%), followed by *Photobacterium* (25.47 ± 9.44%), *Ureaplasma* (6.35 ± 2.82%), *Helicobacter* (4.67 ± 1.75%) and *Vibrio* (4.27 ± 2.68%). *Helicobacter* was present mostly in the stomach (10.91 ± 4.97%).

As for fungal community, 5 phyla, 39 families and 58 genera were detected. At the phylum level (Fig. S1A), Ascomycota (76.60 ± 6.60%) and Basidiomycota (19.34 ± 5.32%) were detected in all the regions with a relatively high abundance. Zygomycota (7.81 ± 7.81%) and Neocallimastigomycota (2.10 ± 2.10%) were just detected in the stomach samples. At the family level (Fig. S1B), Trichocomaceae (18.75 ± 5.08%) and Mycosphaerellaceae (17.14 ± 4.53%) were prevalent along the GI tract. Schizophyllaceae was present in the stomach (5.13 ± 5.13%), hindgut (1.82 ± 1.82%) and feces (1.92 ± 1.92%), but absent in the foregut. Mortierellaceae was exclusively found in the stomach (7.81 ± 7.81%), while Saccharomycetaceae was only in the feces (7.81 ± 7.23%) and Sporormiaceae in the hindgut (7.93 ± 7.93%). At the genus level (Fig. S1C), *Aspergillus*, *Davidiella*, *Alternaria* and *Cryptococcus* were commonly found in all the sites. *Malassezia* was more prevalent in the foregut (9.60 ± 4.59%). *Mortierella* was only detected in the stomach (7.81 ± 7.81%), with *Fusarium* only detected in the foregut (6.18 ± 6.18%) and *Debaryomyces* only detected in the feces (7.81 ± 7.23%).

**Figure 3:**
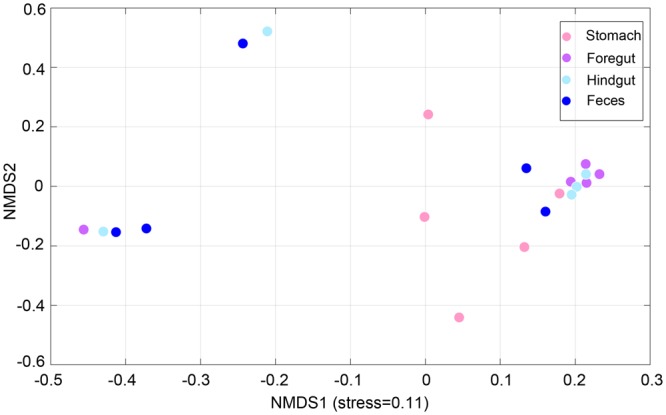
NMDS analysis of all bacterial samples along the GI tract.

To explore dissimilarities between samples along the GI tract, an ordination analysis by using nonmetric multidimensional scaling (NMDS) with a Bray-Curtis distance matrix was performed. There was no evident clustering of bacterial samples by GI regions (Fig. 3). However, when looking at the NMDS analysis closer, bacterial communities in the stomach and foregut tended to be separated while communities in the hindgut and feces did not (Fig. 3). Results of pairwise dissimilarities based on Bray-Curtis distances corroborated the NMDS analysis (Table S1). Furthermore, the microbial community was partitioned by individual subject, especially in the Subject 1 (*P* < 0.05, Table S2). It should be noted that there was marginally significant separation between Subject 5, a porpoise which lived in captivity, and the four other porpoises which lived in the wild (*P* < 0.1, Table S2). As for fungal analysis, GI regions did not contribute to the separation of all the samples significantly (Table S1). Among these five individuals, only slight separation of fungal communities was found in several individual pairs comparison (Table S2).

**Figure 4:**
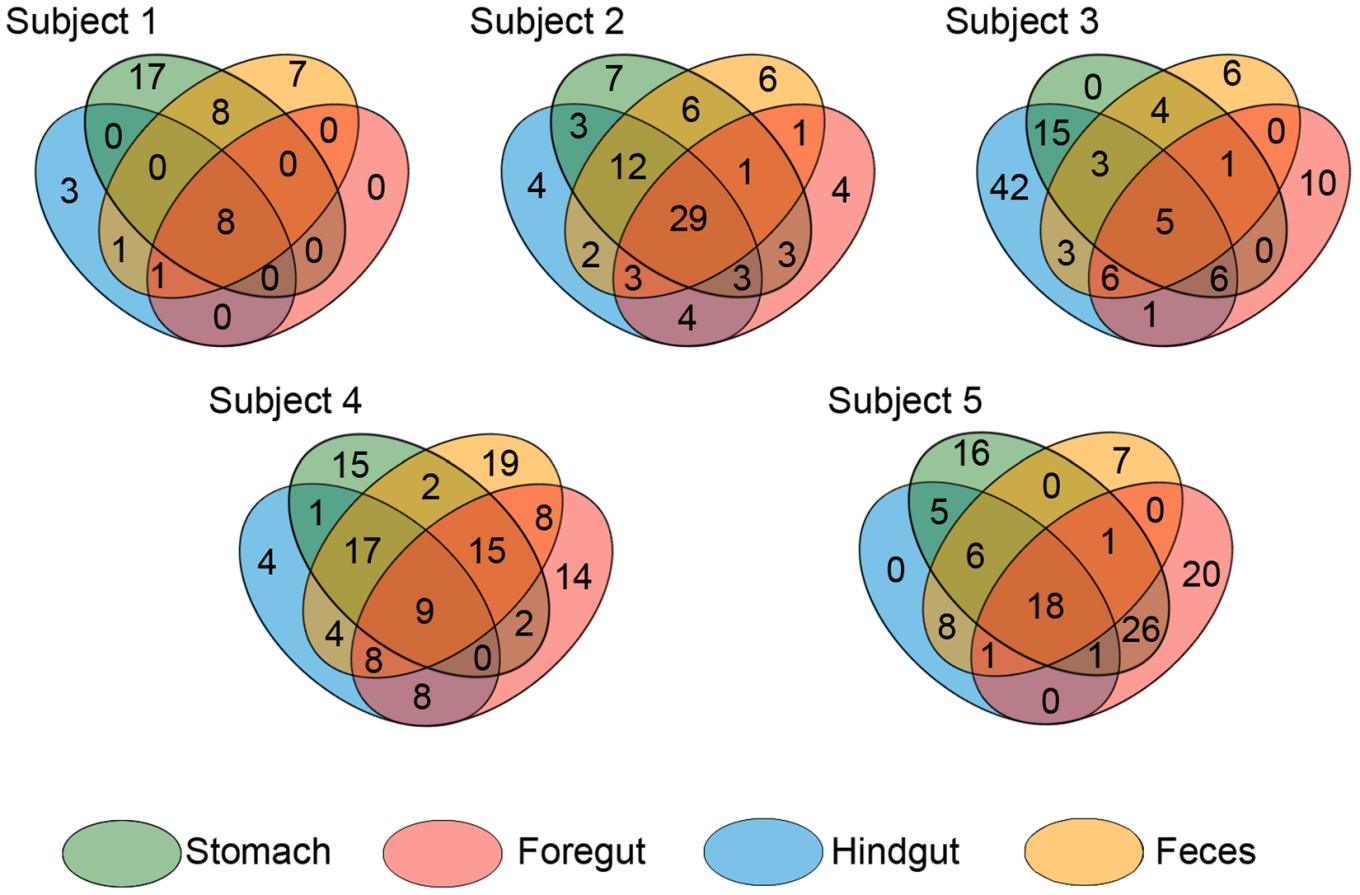
OTU overlap across sampling GI regions. Numbers correspond to unique OTU clusters within a subset.

**Figure 5:**
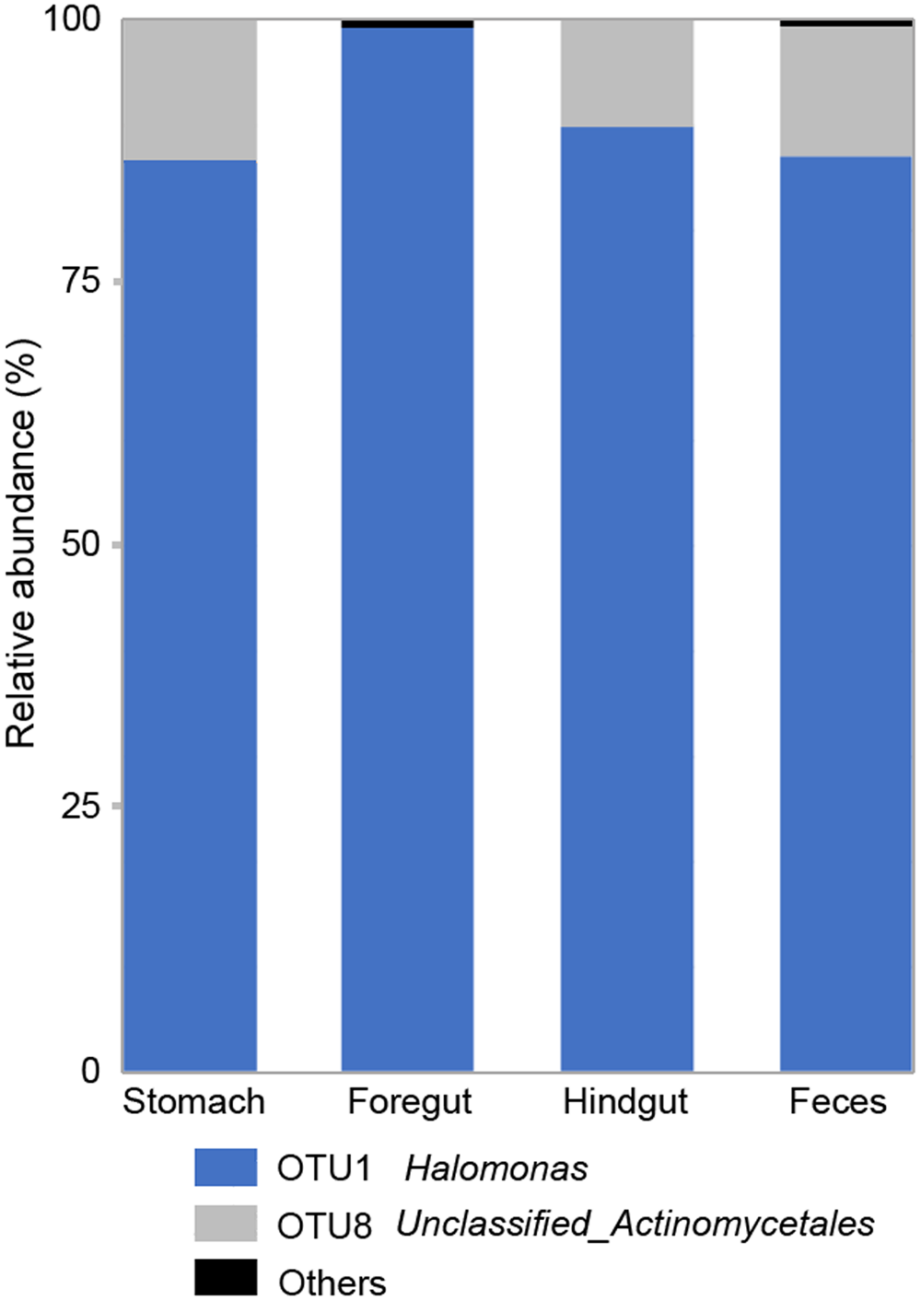
The composition of shared microbiota of Stomach, Foregut, Hindgut and Feces samples. Others: taxa abundance <1%.

For each individual porpoise, overlapping bacterial OTUs for each anatomic region was calculated (Fig. 4). A subset of 5–29 OTUs was present in every sampling region for each individual. The resulting Venn diagrams demonstrated almost consistent overlap patterns for each subject. Hindgut contents and feces had a moderate to high OTU overlap within a subject. There was only one OTU (OTU1, *Halomonas*) which was present in all four regions in the five subjects, accounting for 41.63% of the total sequences (Fig. S3). The different regions shared a similar common microbiota both in amount and in composition (Fig. 5). The stomach, foregut, hindgut and feces samples of these five individuals harbored a small ‘shared’ microbiota (2 OTUs) with OTU1 being the most prevalent in all regions. While for fungi, no overlap was found for each anatomic region from 5 individuals.

## Discussion

Fecal samples have been widely used to study the microbiota of the gastrointestinal tract of cetaceans, especially those animals living in the wild. However, studies on microbiota in the different regions of the GI tract in cetaceans are limited. It is unknown how well fecal samples represent the microbial diversity along the different regions of the cetacean GI tract, and no studies had previously assessed the fungal diversity. These limitations have been overcome to a certain extent in this study by our sampling and sequencing efforts. Our study is the first attempt to characterize the microbiota along the GI tract of the EAFP.

The fecal material of EAFPs were dominated by the phylum Gammaproteobacteria, followed by members of the phyla Fusobacteria, Firmicutes, Tenericutes, Epsilonproteobacteria and Actinobacteria (Fig. 2A). Very few reads were assigned to Bacteroidetes. This distribution was similar to that of bottlenose dolphins^15^ while different from baleen whales which were dominated in a large part by bacteria in the phyla Bacteroidetes and Firmicutes with either very few or no reads assigned to Proteobacteria^13^. As mentioned in the previous study, different gut microbiome profiles in dolphins and baleen whales could mirror their respective dietary niches^15^. Both EAFPs and bottlenose dolphins have a piscivorous diet, which may explain their similarity in GI bacterial communities. It should be noted that Subject 5, the porpoise previously living in captivity, possessed a higher percentage of Firmicutes in the stomach, hindgut and feces than the other EAFPs. In a previous gut bacterial study on freshwater Yangtze finless porpoises, it also found that Firmicutes was more predominant in the fecal material of the captive group than porpoises living in semi-nature conditions^17^. Different living condition (wild and captivity) may help explain the different results between wild and captive EAFPs. The higher percentage of Firmicutes present in the captive EAFPs and Yangtze finless porpoises is noteworthy.

In terms of the fungal community in EAFPs, Ascomycota and Basidiomycota were the two most abundant phyla along the GI tract (Fig. S1A). Ascomycota has been widely distributed in the gut of animals, such as dogs^10^, cats^10^ and giant pandas^22^. The only other study which examined fungal communities in a cetacean, the blue whale, also showed over 99% of the sequences are from the phylum Ascomycota^14^. However, unlike the high proportion of *Metschnikowia* (94.55–98.17%) in the blue whale fecal samples, *Aspergillus*, *Alternaria* and *Cryptococcus* were the predominantly identifiable genera along the GI tract of EAFPs (Fig. S1C). *Aspergillus* species have always been widely studied because of their role in medical, food spoilage and industrial aspects^23^ as well as in human disease^24^. *Alternaria* harbors hundreds of species, some of which have been reported as biocontrol agents against invasive plant species and others as human pathogens^25,26^. *Cryptococcus* is a unique environmental fungus which includes more than three dozen species. However, only *C*. *neoformans* and *C*. *gattii* commonly cause disease in humans and animals^27^. However, the function of these abundant fungi in the GI tract of EAFPs is unknown. Our fungal diversity data highlights the need for further studies on the fungi colonizing EAFPs GI tract and their roles in host health.

Our results obtained from the bacterial and the fungal analysis showed greater diversity in fecal and hindgut samples as well as stomach samples, leaving the least diversity in the foregut samples (Table 1). This probably suggests regional specialization. This trend has also been found in mice^28^. In bottlenose dolphins, a greater bacterial diversity was detected in gastric samples compared to fecal samples^29^. However, the changing pattern in community diversity along the GI tract is not generally found in other hosts. For example, a recent study on rats demonstrated increased community diversity along the digestive tract^19^. In fish, varied patterns including decreased diversity along the turbot (*Scophthalmus maximus*) GI tract was seen^30^. An increased diversity was found along the marine herbivorous fish silver drummer (*Kyphosus sydneyanus*) gut^31^ and similar diversity along the gut of juvenile Atlantic salmon (*Salmo salar L*.)^32^. These resulting differences were probably due to varied sampling methods (content and mucus), host GI traits, and changing autochthonous communities partitioning within the GI tract. However, the factors shaping the GI traits of EAFPs, as well as other cetaceans need to be resolved. This finding indicated that the changing pattern of the GI microbiota diversity of a certain host may not apply to other hosts.

In EAFPs, the stomach seemed to harbor the most distinct microbiota with higher bacterial and fungal diversity detected than in other regions. Gammaproteobacteria and Tenericutes were found prevalent phyla in the forestomach (Fig. 2A). This was similar to the forestomach of bottlenose dolphins with Tenericutes, Bacteroides and Proteobacteria the most abundant^29^. Tenericutes was also found dominant in the stomach of a striped dolphin (*Stenella coeruleoalba*)^33^ as well as the Giant African Snail (*Achatina fulica*)^34^. At the genus level, *Ureaplasma* (from the phylum Tenericutes), *Vibrio* and *Helicobacter* (from the phylum Epsilonproteobacteria) were detected at high abundance in the forestomach (Fig. 2C). *Ureaplasma* was previously detected in the dolphin forestomach at high abundance^29^. As for *Helicobacter*, it has been detected in the stomach and the rectum of dolphins and pinnipeds^29^, as well as in the fecal material of the Yangtze finless porpoise^35^. The stomach samples of EAFPs also harbored a relatively higher percentage of *Mortierella* (from the phylum Zygomycota) than in other regions (Fig. S1C). In a previous study, the authors have attributed the relatively high diversity in the stomach to the existence of a ‘transient microbiota’ from prey species^28^. Also, most of the seawater-associated reads in marine mammal species were found in the dolphin forestomach, suggesting that some dolphins might have ingested seawater just before or at the time of sample collection^29^. In the digestive tract of a timber rattlesnake devoid of digesta, DNA from the prey was detected, which suggests DNA can remain intact in nature^36^. We hypothesized that the higher bacterial diversity found in the forestomach of EAFPs in our study could be from the food ingested, secondary predation. However, the exact mechanism explaining the result of the high abundant of certain microbial groups in the stomach is not yet clear. For example, a previously unclassified bacterial species (belonging to the phylum Tenericutes) was abundant in the forestomach of most dolphins. The authors hypothesized that this species could be involved in the digestion of fish^29^. But further studies are required. Notably, *Halomonas* was shared along the GI regions with quite a large proportion (41.63%)(Fig. 2C). *Halomonas* was identified as one of the most predominant cultivated protease-producing bacteria because it’s chemoorganotrophic^37^, and was once reported dominant in the carnivorous fishes^38^. In addition, *Halomonas* spp. has been shown to be transferred from the dam’s milk to the jejunum and ileum of the newborn sika deer^39^. This suggested that *Halomonas* species might play a significant role in the host’s digestive system and the gut immune system development in hosts. However, this hypothesis needs to be tested in a pure culture approaches.

According to the NMDS analysis, no evident clustering of these samples by GI sites was detected, resulting in a seemingly similar microbial composition among the sites sampled. That different regions shared a similar common microbiota both in amount and in composition supports the above conclusion. Moreover, the inter-individual difference was higher than the intra-individual difference. These observations were contrary to those previous studies which indicated a relatively higher intra-individual difference than inter-individual difference^19,28^. In those studies, subjects were housed in the same location with the same diet, which may help control environmental factors. While four wild EAFPs sampled in our study were free-ranging, living in the Bohai/Yellow Sea before their death, and the one captive porpoise was housed indoor for more than one year. Differences in habitats and food intake among our animals sampled in the present study may contribute to the higher inter-individual difference of the GI microbiota. Our results might show that the GI microbiota in wild hosts may be more complicated than our existing knowledge which was only based on the captive animals. Extensive studies on the GI microbiota from more wild hosts are needed. However, in spite of similar microbial composition among these sites, physicochemical conditions of the different sites seemed to aid in shaping the microbiota. For example, strict anaerobic bacteria belonging to members of Clostridia (Class), Fusobacteria (Class), and Ruminococcaceae (Family) were enriched in the hindgut and fecal samples where less oxygen is available. In this respect, fecal samples are not representative of microbiota along the entire GI tract of EAFPs.

Many infectious diseases have been reported to impact cetacean populations^40^ while seldom have these diseases been detected in EAFPs. *Helicobacter* spp. are able to naturally colonize the lower intestinal tract of an animal host and can cause gastritis and gastric cancer^41,42^. In the present study, sequences belonging to the genus *Helicobacter* were found along the GI tract, most notably in the stomach, and to a lesser extent in the fecal material. *Vibrio* was enriched in the stomach, but was also detected in the feces, hindgut and foregut. *Vibrio* species, including *Vibrio harveyi*, are associated with diseases in fish^43,44^. In addition, *Vibrio damsela* and *Vibrio alginolyticus* have been reported to infections in dolphins^45,46^. Even though the clinical relevance of these bacterial species in EAFPs remains unclear, the detection of these potentially pathogenic bacteria groups in fecal samples indicated the necessity of fecal monitoring in EAFPs.

To our knowledge, this is the first endeavor to explore the entire bacterial and fungal community along the EAFP GI tract. Only five individuals were included in this study. Increasing the sample size will yield additional data. However, these animals are protected and they cannot be sacrificed for experimental purposes. In future, more studies will be conducted using samples from animals which immediately died of natural or accidental causes. Furthermore, we have only examined four sites along the GI tract. Cetaceans harbored a very long GI tract which is much longer than the body length. In future multi-locus sampling along the GI tract, including the three stomach compartments, will be done to better characterize the whole GI tract microbiome in cetaceans.

This study was the first high-throughput sequencing analysis of the GI microbiota of EAFPs. Our findings provide a baseline for understanding the complexity of EAFP GI microbial ecology and suggests that fecal monitoring is still of importance because of their relatively higher microbial diversity and presence of potential pathogens. To our knowledge, the present work is the first study on the microbiota along the GI of a cetacean species. This study will lay the foundation for better understanding of the GI microbial communities in other cetaceans.

## Materials and Methods

### Sample collection and DNA extraction

In total, five EAFPs were collected in Penglai City, China. Subject 1–4 were accidentally caught in gill nets and discovered within a few hours after death by local fishermen near the Bohai/Yellow Sea in May 2015. The region where the animals were found is the same area as cited in a recent study by Wan *et al*.^47^. Subject 5 was living in captivity at Penglai Sea World. In May 2015 the porpoise died suddenly for unknown reasons. No antibiotics or other medications were given while the porpoise lived in captivity. All five carcasses were immediately transported to an autopsy suite at Penglai Sea World. Basic information for all the sampled EAFPs is showed in Table 2. All samples were collected under sterile conditions. The stomach (forestomach), foregut (from the pyloric ceca to the middle of the intestine), hindgut (distal half of the intestine) and fecal (rectal) samples were removed by dissection. The content of each digestive segment was gently squeezed out and homogenized separately. All samples were preserved in liquid nitrogen until DNA extraction. Necropsy and sampling were conducted in accordance with the Regulations of the People’s Republic of China for the Implementation of Wild Aquatic Animal Protection (promulgated in 1993), adhering to all ethical guidelines and legal requirements in China. The protocol of this study was approved by the Institutional Review Board of the Institute of Hydrobiology, Chinese Academy of Sciences.

**Table 2:** Basic information for all the five East Asian finless porpoises sampled in this study.

Animal ID	Sex	Body length (cm)	Weight (kg)	Living environment
Subject 1	F	138	35.2	Free-ranging
Subject 2	F	120	26.4	Free-ranging
Subject 3	M	105	21.5	Free-ranging
Subject 4	F	135	34.2	Free-ranging
Subject 5	F	131	29.5	Indoor-captive

Note: Body length, straight length from the snout to fluke notch. F, female; M: male.

Total genomic DNA from each sample was extracted using the ZR Fecal DNA Kit (Zymo Research Inc., CA, USA) according to the manufacturer’s instructions. Extracted DNA was quantified by a Nanodrop 2000 spectrophotometer and stored at −80 °C until subsequent procedures.

### PCR amplification and high-throughput sequencing

PCR amplifications were carried out using the TransStart FastPfu DNA Polymerase on the ABI GeneAmp^®^ 9700 PCR system. For bacteria, PCR amplification of the 16S rRNA gene was performed in three replicates based on the study by using the universal primers 341 F (5′-CCT AYG GGR BGC ASC AG-3′) and 806 R (5′-GGA CTA CNN GGG TAT CTA AT-3′) specific for the V3-V4 regions^48^. The PCR assays were carried out in triplicates as follows: 20 μl reaction solutions with 4 μl FastPfu Buffer, 2 μl dNTPs (2.5 mM), 0.8 μl of each primer (5 μM), 0.4 μl FastPfu Polymerase, and 10 ng template DNA. The PCR conditions for bacterial DNA amplification were as follows: 95 °C for 5 min, followed by 27 cycles of 95 °C for 30 s, 55 °C for 30 s, 72 °C for 45 s, and a final extension of 72 °C for 10 min. Subsequently, we purified PCR products with a DNA gel extraction kit (Axygen, China). Sample libraries were constructed by adding individual 6-base barcode sequences to DNA fragments of each sample, and then ligating truseq adapters onto PCR products. Libraries DNA were purified using AMPure XP beads (CA, USA) to remove short fragments. Purified libraries were then quantified using Qubit and quality detected on an Agilent Technology 2100 Bioanalyzer. For fungi, ITS-1 regions were amplified using universal primers ITS1F (5′-CTT GGT CAT TTA GAG GAA GTA A-3′) and ITS2 (5′-GCT GCG TTC TTC ATC GAT GC-3′)^49^. The number of initial PCR cycles was adjusted to 29 cycles, and all other steps were same as with the bacterial DNA libraries construction. Amplicon sequencing was carried out on the Illumina HiSeq 2500 platform which generated 250 bp paired end raw reads.

### Sequence processing and statistical analysis

The amplicon sequence data was analyzed using an internal pipeline Amplicon Sequencing Analysis Pipeline (ASAP, version 1.3). The HiSeq sequences were first subjected to quality check with FastQC (version 0.11.5). The paired-end sequences were then merged based on the 3′ overlap using PEAR (version 0.9.10)^50^ with a quality score cutoff of 20, minimum assembled length of 200, maximum assembled length of 400 and minimum overlap length of 50 bp. The program split_libraries_fastq.py of QIIME packages (version 1.9.1)^51^ was used to assign reads to samples (demultiplexing) based on the barcodes with the maximum barcode error of 0 and trimming quality score cutoff of 20. Primer sequences (forward and reverse) were trimmed. Sequences of library splitting of multiple sequencing rounds (two rounds in this study) were merged. Dereplication was performed using USEARCH (version 9.2.64)^52^ with the command fastx_uniques (with the option of -sizeout for sequence abundance output). Operational Taxonomic Units (OTUs) were clustered using UPARSE (command -cluster_otus of USEARCH)^53^ with OTU identity threshold of 0.97 and singletons and chimeric sequences were removed during this process. OTU table was made using command of -usearch_global of USEARCH. The representative sequences of OTUs were classified using RDP Classifier (16S: training set 16, June 2016; ITS: trainset fungalits_warcup, July 2016)^54^ with confidence cutoff of 0.8. OTUs assigned to Chloroplast (at Order level) were removed. The representative sequences of OTUs were used to construct the phylogenetic tree. Sequences were aligned using MAFFT (version 3.8.31)^55^ and alignments were filtered using Gblocks (version 0.91b)^56^ with option of −t = d, −b4 = 3 and −b5 = h. FastTree^57^ was used to construct phylogenetic tree with the filtered alignment. Bacterial and fungal sequences have been deposited in the GenBank Sequence Read Archive under the accession numbers SRP106577 and SRP107160, respectively.

The richness indices were based on the number of OTUs and Chao 1 index detected in each GI region or each animal. The α-diversity was represented as Shannon’s index. Both Chao 1 and Shannon indices in different number of sequences sampled were calculated based on the OTU table using programs of QIIME through the pipeline ASAP mentioned above. Rarefaction curves based on the Shannon indices in different number of sequences sampled were plotted using Qiime. The β-diversity was calculated based on Bray-Curtis distance using Vegan (v.2.4–6) and Ieggr (v.3.1) packages in R software (v.3.4.3) (R Core Team, 2017). Pairwise comparisons of richness (Chao1) and α-diversity (Shannon) indices between different GI regions as well as different individuals were performed using Wilcoxon rank sum test in R software (v.3.4.3) (R Core Team, 2017). *P* values were corrected using false discovery rate. Significance was accepted at *P* < 0.05. Nonparametric multivariate statistical analyses, including Analysis of Similarity (Anosim), Multiresponse Permutation Procedures (MRPP) and permutational multivariate analysis of variance (Adonis) based on Bray-Curtis distance were used to determine the dissimilarity of microbial profiles among individuals and GI regions, using the Vegan (v.2.4-6) and Ieggr (v.3.1) packages in R software (v.3.4.3) (R Core Team, 2017).

## Electronic supplementary material

Supplementary Information-GI tract-second revision
