# Supplementary material for: Microbial communities in different regions of the gastrointestinal tract in East Asian finless porpoises (Neophocaena asiaeorientalis sunameri)
Source: Sci Rep. 2018 Sep 20;8:14142. doi: 10.1038/s41598-018-32512-0 (PMC6147976; doi:10.1038/s41598-018-32512-0)
Supplement: Supplementary file 1 — Supplementary Information-GI tract-second revision [file 41598_2018_32512_MOESM1_ESM.pdf]

Microbial communities in different regions of the gastrointestinal tract in East Asian finless porpoises (*Neophocaena asiaeorientalis sunameri*)

Xiao-Ling Wan[1,2], Richard William McLaughlin[1,3], Jin-Song Zheng[1,*], Yu-Jiang Hao[1], Fei Fan[1], Ren-Mao Tian[4], Ding Wang[1]

[1] The Key Laboratory of Aquatic Biodiversity and Conservation of the Chinese Academy of Sciences; Institute of Hydrobiology, Chinese Academy of Sciences, Wuhan 430072, China;

[2] University of Chinese Academy of Sciences, Beijing 100039, China;

[3] General Studies, Gateway Technical College, Kenosha, WI 53144, USA;

[4] Institute for Environmental Genomics, University of Oklahoma, Norman, OK 73019, USA.

***Correspondence to:**

Jin-Song Zheng

Fax: 86-27-87491267

Email: zhengjinsong@ihb.ac.cn

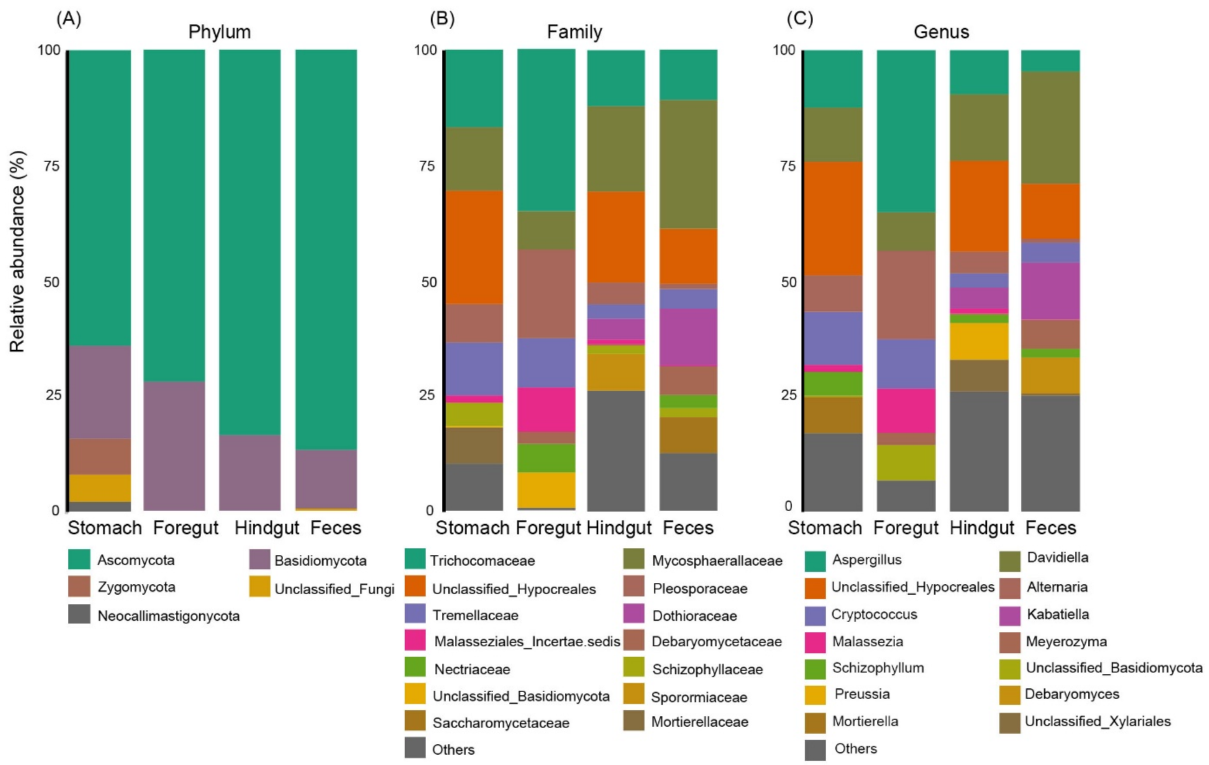

Figure S1. Fungal composition along the GI tract of East Asian finless porpoises at the Phylum (A), Family (B) and Genus (C) levels.

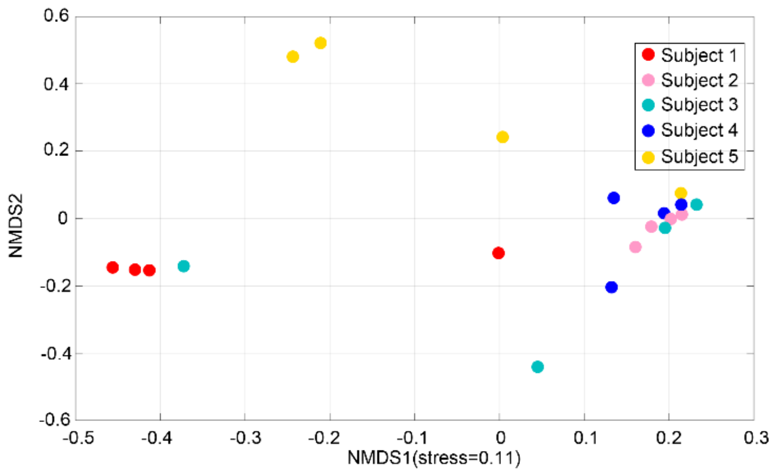

Figure S2. NMDS analysis of all bacterial samples in five East Asian finless porpoises.

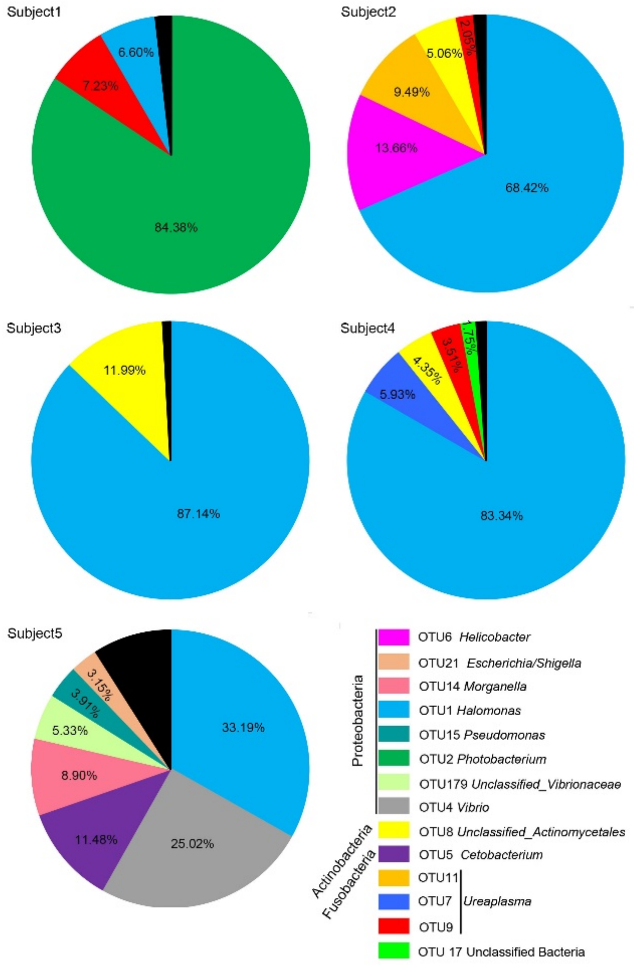

Figure S3. Shared microbiota within different GI regions. Others: taxa abundance <1%.

Table S1. Non-parametric multivariate dissimilarity tests of bacterial and fungal community composition along the GI tract. **Anosim**, analysis of similarity; MRPP, multi-response permutation procedures; **Adonis**, permutational multivariate analysis of variance using distance matrices. Results presented are based on distance matrices calculated with Bray-Curtis index. *P* values <0.1 are in bold.

	Group1	Group2	Anoism		MRPP		Adonis	
			R	*P*	*δ*	*P*	*F*	*P*
Bacteria	**Stomach**	**Foregut**	0.134	**0.078**	0.639	**0.027**	2.216	**0.029**
	Stomach	Hindgut	-0.062	0.720	0.804	0.346	0.974	0.489
	Stomach	Feces	0.070	0.293	0.843	0.349	1.151	0.412
	Foregut	Hindgut	-0.068	0.680	0.600	0.538	0.476	0.514
	Foregut	Feces	0.080	0.226	0.640	0.178	1.560	0.183
	Hindgut	Feces	-0.140	0.800	0.810	0.586	0.474	0.649
Fungi	Stomach	Foregut	-0.186	0.99	0.975	0.975	0.576	0.983
	Stomach	Hindgut	-0.150	0.931	0.953	0.990	0.483	0.993
	Stomach	Feces	-0.026	0.555	0.911	0.694	0.838	0.65
	Foregut	Hindgut	-0.022	0.560	0.962	0.728	0.880	0.736
	Foregut	Feces	0.084	0.198	0.920	0.295	1.112	0.279
	Hindgut	Feces	-0.062	0.592	0.900	0.816	0.671	0.800

Table S2. Non-parametric multivariate dissimilarity tests of bacterial and fungal community composition among five East Asian finless porpoises. **Anosim**, analysis of similarity; MRPP, multi-response permutation procedures; **Adonis**, permutational multivariate analysis of variance using distance matrices. Results presented are based on distance matrices calculated with Bray-Curtis index. *P* values <0.1 are in bold.

	Group1	Group2	Anoism		MRPP		Adonis	
			R	*P*	*δ*	*P*	*F*	*P*
Bacteria	**Subject1**	**Subject2**	0.875	**0.027**	0.418	**0.037**	0.418	**0.037**
	**Subject1**	**Subject3**	0.427	**0.048**	0.673	0.180	0.673	0.180
	**Subject1**	**Subject4**	0.750	**0.026**	0.529	**0.071**	0.529	**0.071**
	**Subject1**	**Subject5**	0.885	**0.024**	0.616	**0.027**	0.616	**0.027**
	Subject2	Subject3	-0.031	0.524	0.607	0.277	0.607	0.277
	Subject2	Subject4	-0.042	0.657	0.463	0.441	0.463	0.441
	**Subject2**	**Subject5**	0.552	**0.058**	0.550	**0.052**	0.550	**0.052**
	Subject3	Subject4	-0.083	0.759	0.719	0.695	0.719	0.695
	**Subject3**	**Subject5**	0.490	**0.063**	0.806	0.208	0.806	0.208
	**Subject4**	**Subject5**	0.438	**0.078**	0.662	0.128	0.662	0.128
Fungi	Subject1	Subject2	0.084	0.198	0.920	0.295	1.112	0.279
	Subject1	Subject3	-0.062	0.592	0.900	0.816	0.671	0.800
	Subject1	Subject4	-0.021	0.574	0.900	0.529	0.738	0.671
	**Subject1**	**Subject5**	0.229	**0.089**	0.803	0.106	1.713	0.101
	Subject2	Subject3	0.026	0.441	0.914	0.200	1.481	0.175
	Subject2	Subject4	0.083	0.192	0.910	0.202	1.261	0.200
	Subject2	Subject5	-0.016	0.435	0.817	0.303	1.097	0.332
	**Subject3**	**Subject4**	0.229	0.128	0.928	**0.064**	1.344	**0.055**
	Subject3	Subject5	-0.094	0.892	0.924	0.689	0.798	0.723
	**Subject4**	**Subject5**	0.401	**0.028**	0.831	**0.028**	2.207	**0.029**
